# Pulp Changes Secondary to Orthodontic Forces: A Review of Literature

**DOI:** 10.7759/cureus.40573

**Published:** 2023-06-17

**Authors:** Mustafa Hussein Alattas

**Affiliations:** 1 Conservative Dental Science and Endodontics, Qassim University, Buraydah, SAU

**Keywords:** pulpal response, dental pulp volume, pulpal blood flow, orthodontic forces, pulp changes

## Abstract

This review article encompasses the literature pertaining to changes in the dental pulp subsequent to forces exerted secondary to orthodontic treatment. The review was conducted at the College of Dentistry, Qassim University, Saudi Arabia, from October 2022 to February 2023. A literature search was conducted on PubMed, Embase, The Cochrane Library and Google Scholar for articles from 2000 to 2023. Keywords and MeSH terms used were ‘orthodontic forces’, ‘pulp changes’, ‘dental pulpal changes’, and ‘pulp volume’. Two reviewers went through the titles. After removing irrelevant and duplicate articles, abstracts were assessed for relevant articles. Finally, the reviewers analyzed full-text articles, and a total of five articles including four randomized controlled trials and one retrospective study were selected. It was concluded that managing and minimizing injury to the pulp or supporting tissues is important when using orthodontic mechanics. In order to do so, clinicians must thoroughly understand any change in the pulpal tissue following the orthodontic treatment. Orthodontic tooth movements cause inflammatory changes in the tooth pulp and periodontal ligament which are directly related to the amount, direction, and time duration of force used. Long-term pulp blood flow analysis shows that even though there is a transient uptrend in the flow of blood after the removal of the orthodontic force, it reverts to normal levels three months later. However, pulp volume has been reported to decrease, more prominently in anterior teeth, as orthodontic forces stimulate the pulp to produce tertiary dentin.

## Introduction and background

Orthodontic treatment is based on applying forces on teeth for a predetermined duration, ranging from months to years. The tissue cells in the periodontium have been shown to undergo molecular alterations as a consequence of this force [1]. Force application causes an initial inflammatory response in the periodontal ligament (PDL) during the early stages of tooth movement in orthodontics. This stage involves vasodilatory changes along with the transport of white blood cells from the blood into the periodontal tissue [2]. An interaction is then initiated between these migrated cells and the local cells of the periodontium. In turn, a variety of regional biochemical signal molecules including cytokines are produced. Within a day or two, this is followed by a chronic inflammatory response in which fibroblasts, endothelial cells, osteoblasts, and alveolar bone marrow cells replace the acute phase. In this stage, leukocytes keep migrating and modulate the remodeling process [3].

Cell destruction, inflammation, and wound healing are all aspects of the pulpal response to orthodontic treatment that may have a negative impact on the tooth pulp. Published histological studies show that pulp exhibits reactions ranging from circulatory vascular stasis to necrosis in response to orthodontically exerted pressures [4]. Orthodontically exerted forces are associated with a decrease in the respiratory rate of tissue cells, along with a decrease in the activity of alkaline phosphatase, and other processes, including apoptosis, aspiration, vacuolization of odontoblasts, and tissue injury. An increase in micro-vessels and the discovery of angiogenic alterations in human dental pulp point to higher levels of angiogenic growth factors in the pulpal tissue. Many variables, including the type/direction of movement, and the distribution, intensity, and duration of the force, affect how much the tooth pulp changes. The pulp conditions may also be impacted by the tooth's inherent characteristics, including age, past orthodontic treatment, and trauma history [5].

Recently, researchers have studied a number of aspects of how orthodontic forces affect the dental pulp, including blood flow in the pulp, response to tooth sensitivity testing, the expression/activity levels of various enzymes and neuropeptides, in addition to changes in the histology and morphology of the tissue [6]. Blood flow in the pulp is a sign of the vitality of the pulp. The pulpal sensory responses and tooth sensitivity may decline due to reduced pulpal blood flow or brief ischemia. In such cases, indirect testing may be utilized to determine pulpal vitality, since the pulp is enclosed in a calcified cavity [7]. Because noninvasive procedures are easy for clinical use, they can be used during orthodontic therapy to check the state of the pulp. In order to repeatedly clinically measure the flow of blood and conduct sensitivity tests without causing damage to the tissues, laser Doppler flowmetry and electric/thermal pulp tests (EPTs) are performed [8]. Clinicians must therefore be aware of the clinical symptoms and indicators that could indicate pulp changes brought on by an orthodontic force.

When an orthodontically generated force is exerted on the pulp tissue, it results in the release and accumulation of inflammation-associated mediators. This causes odontoblasts and associated cells to respond in a resorptive or reparative manner, which can result in either resorption or tertiary dentin deposition [9]. Neuropeptide production calcitonin gene-related peptide (CGRP) and substance P (SP) are higher in inflamed pulpal tissue, in comparison to normal pulpal tissue [10]. Such occurrences may lead to internal alterations in the pulp cavity. The dentist should have imaging diagnostic tests during patient treatment to monitor these alterations [5].

Thus, a thorough analysis of current research from an orthodontic perspective was deemed clinically instructive. When using orthodontic forces to manage and reduce injury to the pulpal and periodontal tissue, clinicians must have a thorough understanding of these changes. The present study aimed to review the studies assessing pulp response to orthodontic forces.

## Review

Materials and methods

A review of the literature was conducted using the following databases: Embase, The Cochrane Library, MEDLINE-PubMed, and Google Scholar. A search of the gray literature was also done. All searches were made from 2020 to 2023. The keywords and MeSH terms used were ‘orthodontic forces’, ‘pulp changes’, ‘dental pulpal changes’, and ‘pulp volume’. The Boolean operators ‘OR’ and ‘AND’ were used for a thorough literature search. Only relevant randomized controlled trials and cohort studies were included in the review. Case reports, case series, conference proceedings, gray literature, and animal studies were excluded.

Two phases of study selection were carried out. Phase 1 involved the review of all identified references' titles and abstracts by two reviewers. Both the reviewers applied the same strategy to analyze the articles in phase 2. The reviewers individually went through the chosen studies to look for potentially pertinent papers. Any disagreements were settled between them, and a third reviewer was consulted if there was no agreement.

In a pilot study, a data collection form was created and assessed. After training, the two reviewers extracted crucial information from the chosen research, and they resolved any conflicts among themselves. Both study and sample characteristics were recorded. Moreover, orthodontics-related variables and details of the methods used were also collected, along with outcome assessment and main findings.

Results and discussion

**Figure 1:**
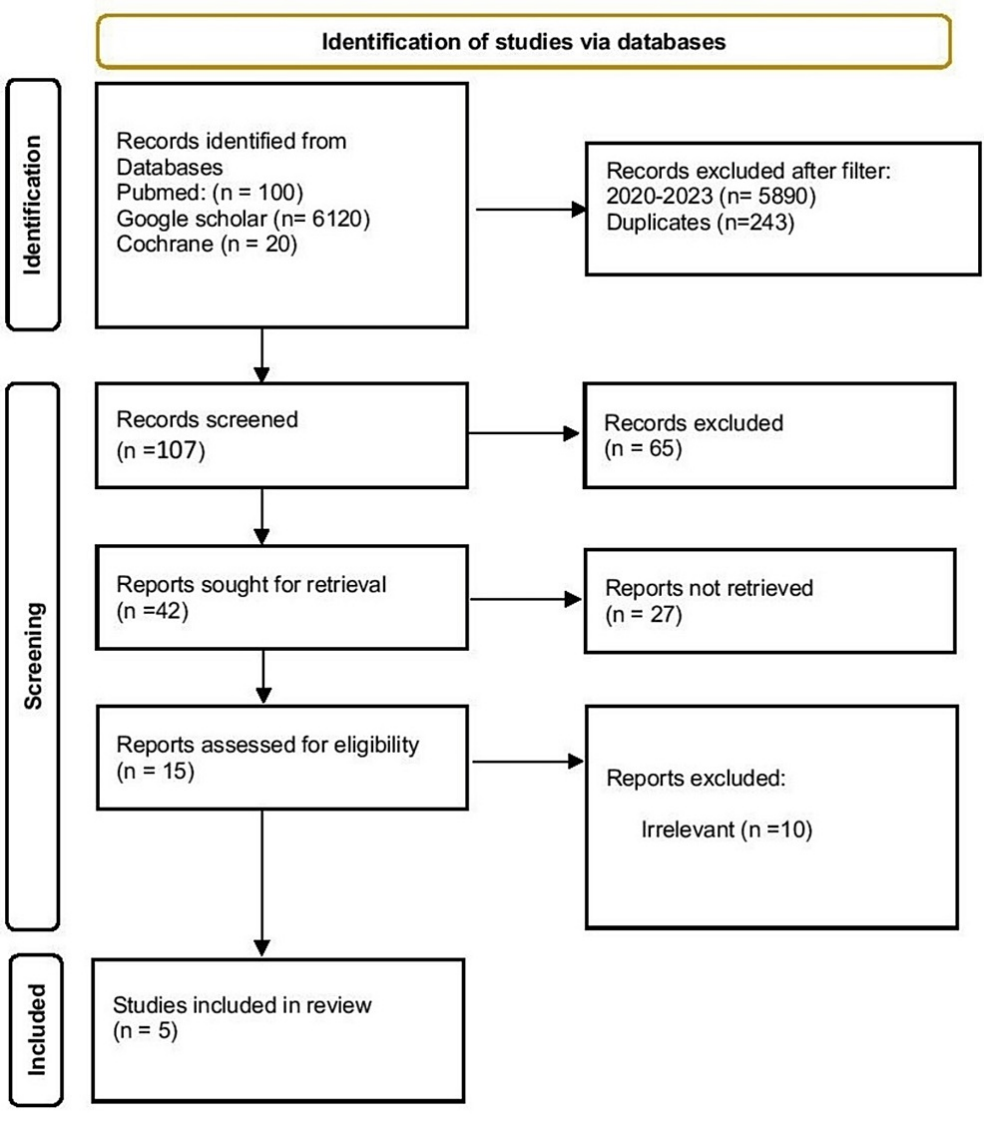
PRISMA Flowchart of the Selection Process PRISMA: Preferred Reporting Items for Systematic Reviews and Meta-Analyses.

The selection process resulted in a final list of five articles for the final evaluation. Of these, four were randomized clinical trials and one was a retrospective study (Figure 1). Orthodontic forces were found to be associated with increased flow of blood, reduction in pulpal volume, more inflammation, and CGRP expression. However, no increase in caries or root canal treatment incidence was reported during orthodontic treatment [11-15].

Hatrom et al. studied the changes in pulp volume and root length of the upper anteriors requiring en masse retraction with and without piezo-orthodontics. They observed a reduction in pulp volume following the application of retractive forces using three-dimensional analytical software [14]. Guo et al. examined any change in pulp blood flow (PBF) in anteriors using laser Doppler flowmetry (LDF) after retraction [13]. The results showed that the teeth ready for debonding showed significantly higher levels of PBF compared to non-orthodontic teeth at one month. There was a transient rise in PBF levels after the appliance was removed. However, the levels went back to normal in about three months [13]. Caviedes-Bucheli et al. assessed the CGRP levels after subjecting premolars that were planned for extraction to different intensities of orthodontic forces. They observed that levels of CGRP were proportional to the amount of force applied; the teeth subjected to greater force showed greater expression of CGRP by radioimmunoassay [12]. Mago et al. evaluated the effect of orthodontic forces applied to upper premolars [15]. A histological analysis was done (inflammation, fibrous tissue, and vascular dilatation). Histological analysis revealed that the application of intrusive forces on maxillary anterior teeth resulted in mild inflammation and vascular dilatation after seven days, whereas the inflammation was reversed, and fibrous tissue was observed after one month of the force application. However, no histological changes were observed in the pulp from the mild intrusive force [15]. Finally, a retrospective study by Alanazi et al. evaluated the incidence of root canal treatment and deep caries after orthodontic treatment. Results showed no change in the incidence of caries or root canal treatment in orthodontically treated teeth [11], as illustrated in Table 1.

**Table 1:** Summary of Included Articles RCT: randomized controlled trial; PM: premolars; BL bilateral; CBCT: cone beam computed tomography.

Author, Year, Type	Aim	Method	Result	Conclusion
Hatrom et al., 2021 [14] RCT	To compare pulpal volume changes in the upper anteriors between teeth retracted with piezo-orthodontics (G1) and control (G2, normal retraction).	Inclusion: individuals selected for upper PM extractions and retraction (BL). Random allocation done. Pulpal volume and changes in root length measured.	Sample = 23 (12 G1, 11 G2). Significant reduction in pulpal volume in all patients (p<0.01). No significant inter-group difference. Pulpal volume moderately correlated with right canine root length (r=0.44; p=0.034).	Piezo-orthodontics has a similar effect on pulpal volume as conventional orthodontics. Pulpal volume changes not associated with root resorption rates.
Guo et al., 2022 [13] RCT	To examine pulp blood flow (PBF) in upper and lower anteriors using laser Doppler flowmetry (LDF) after orthodontically induced retraction. To assess any association between root resorption and age on PBF changes.	Sample: 50 bimaxillary protrusion cases (25 for debonding G1 and 25 controls for C). PBF of upper and lower anteriors measured at day 1 (T1), 3 months (T2) and after removing appliance (T3). Pre- and post-orthodontic treatment CBCT scans used for measuring root resorption.	No inter-group difference in PBF at T1 and T3. Higher PBF found in G1 at T2 (maxillary lateral incisor p=0.048, maxillary central incisor p=0.01, mandibular lateral incisor p=0.021). No difference between sub-groups found.	PBF in upper anteriors found to have a transient rise post-orthodontic device removal and then transitioned to pre-treatment levels after 90 days. Changes pronounced among adolescents. PBF in anteriors is not associated with root resorption.
Caviedes-Bucheli et al., 2022 [12] RCT	To assess the impact of moderate-severe orthodontically generated forces on calcitonin gene-related peptide (CGRP) expression in normal and any potential association with the pulpal tissue.	Sample: 90 PDL samples extracted from healthy PMs. Random allocation into three groups: controls: untreated teeth (C); moderate force (G1): 56 g on PMs over 24 hrs; and severe force (G2): 224 g on PMs over 7 days. After processing PDL samples, CGRP measurements taken using radioimmunoassay.	Highest CGRP levels in G2. The lowest CGRP values were for C. Inter-group differences not significant.	Orthodontically generated forces result in higher CGRP expression in PDL, CGPR being associated with the applied force. This affects the pulpal response to various forces applied.
Mago et al., 2020 [15] RCT	To assess the impact of orthodontically generated forces on pulpal tissues.	Sample: 50 patients for upper first PM orthodontic extraction. Two groups: G1: 16 × 22 steel wire cantilever spring used for applying intrusive forces on maxillary first PMs; and G2 controls: histological changes were compared after 7 days and 1 month.	Inflammation: At 7 days, mild inflammation was seen in both groups (G1 30%; C 20%). No inflammation at one month. Fibrous tissue: None at 7 days; Mild fibrous tissue at 1 month in C (62%). Vascular dilatation: Moderate changes at 7 days (G1 38%; C 40%); at 1 month, 40% dilatation in both groups.	No obvious histological changes were observed in pulp from the mild intrusive force in either group.
Alanazi et al., 2022 [11] Retrospective Study	To ascertain the frequency of endodontic treatment and occurrence of caries affecting the vitality of pulpal tissue secondary to orthodontic appliance-generated forces.	Sample: 100 patient files retrieved (G1: 50 conventional orthodontics; G2: 50 clear alignment). Age = 14-40 years. Pre- and post-treatment radiographic images showed increased occurrence of caries and endodontic treatment.	Caries occurrence was similar between Invisalign and conventional orthodontic treatment.	Caries occurrence rate of 24% observed among all groups. No inter-group difference was found.

Applying an external force to teeth as part of orthodontic mechanics causes the teeth to shift within the alveolar bone. In cases where forces are within a physiologically permissible range, a physiological response results in the pulpal and periodontal tissues without having a deleterious long-term effect [16]. Since the visual assessment of any morphological changes in the tissue is only possible after extracting the tooth, clinicians need to be aware of the clinical symptoms of pulp alterations.

When it comes to identifying vital teeth, laser Doppler flowmetry is highly accurate and outperforms electric and thermal tests. Many recent studies use LDF to assess PBF during orthodontic treatment. According to Ersahan and Sabuncuoglu, the PBF drop that occurred while intruding the upper first molar was reversible [17]. According to Abu Alhaija and Taha, there was no discernible difference when normal and self-ligating fixed orthodontic appliances were compared in terms of PBF changes; both decreased within 48 hours, increased after a week, and then reverted to their previous levels after a month [18]. All of this research revealed that the PBF frequently decreases briefly before quickly returning to its baseline level many weeks later. During the application of orthodontic forces, the compression of periapical vessels may lead to a brief disruption of PBF, which is linked to a drop in oxygen tension and raises the likelihood of injuring the pulpal cells. Since the PBF observation times in the trials were so brief, their findings only capture the immediate impact of orthodontic management. Pulp vitality may be compromised if the force is exerted for an extended duration causing the anterior teeth to move longer distances. Guo et al. examined the PBF in the maxillary and mandibular anterior teeth after orthodontic retraction. A month after the removal of the appliance, Guo et al. observed an intriguing transitory rise in PBF in upper anteriors, which was likely brought on by the relief of vascular compression brought on by the movement of the teeth [13]. Following the release of orthodontic force, blood vessels momentarily dilate; nevertheless, when pulp vessels regenerate, the apical circulation eventually returns. About three months after the removal of the appliance, the PBF in the upper teeth had recovered to normal levels [13].

The relationship between dental pulp volume and root resorption and orthodontic tooth movement's cause and effect is still difficult to understand [19]. Several ways that pulpal tissue has been observed to respond to orthodontic treatment have been documented, but generally speaking, pulpal tissue responds by producing tertiary dentin following its stimulation [3]. The volume of the pulp may be negatively impacted by these repairs. Hatrom et al. observed that after receiving orthodontic treatment, the anterior teeth's pulp volume underwent a significant decline [14]. Their findings were attributed to the pulp's protective response to an irritable stimulus brought on by an orthodontic tooth, which resulted in the deposition of tertiary dentin and a reduction in pulpal volume. They came to the conclusion that following orthodontic treatment, the pulpal volume in the anteriors reduces significantly. Inflammatory effects and changes in the pulp associated with tooth movement secondary to orthodontic forces are plausibly responsible for this decrease in pulp volume [14]. These changes may have mildly irritated the layer of odontoblasts circumferential to the pulpal tissue, leading to increased levels of the inflammatory mediator, which in turn further stimulate odontoblast and odontoblast-like cells. This leads to tertiary dentin being deposited leading to a decrease in the pulpal volume [20].

## Conclusions

Orthodontic movement leads to inflammation in the tooth pulp and PDL which is associated with the amount, direction, and duration of force used. When stimulated by mechanical stress, C-fibers release CGRP, which can influence blood flow and vascular tone, which triggers the rapid and widespread entry of immune-mediated cells and other mediators of inflammation. The orthodontic forces may cause a transient inflammatory response in the initial stages. However, these changes are reversible, and hence, no long-term detrimental effect on dental pulp is observed.
